# Carbon stock growth in a forest stand: the power of age

**DOI:** 10.1186/1750-0680-2-4

**Published:** 2007-04-26

**Authors:** Georgii A Alexandrov

**Affiliations:** 1 Office for Global Environmental Database, Center for Global Environmental Research, National Institute for Environmental Studies, Onogawa 16-2, Tsukuba, Ibaraki 305-8506, Japan; 2 Laboratory of Mathematical Ecology, Institute of Atmospheric Physics, Russian Academy of Sciences, Pyzhevsky Per. 3, Moscow, 109017, Russia

## Abstract

**Background:**

Understanding the relationship between the age of a forest stand and its biomass is essential for managing the forest component of the global carbon cycle. Since biomass increases with stand age, postponing harvesting to the age of biological maturity may result in the formation of a large carbon sink. This article quantifies the carbon sequestration capacity of forests by suggesting a default rule to link carbon stock and stand age.

**Results:**

The age dependence of forest biomass is shown to be a power-law monomial where the power of age is theoretically estimated to be 4/5. This theoretical estimate is close to the known empirical estimate; therefore, it provides a scientific basis for a quick and transparent assessment of the benefits of postponing the harvest, suggesting that the annual magnitude of the sink induced by delayed harvest lies in the range of 1–2% of the baseline carbon stock.

**Conclusion:**

The results of this study imply that forest age could be used as an easily understood and scientifically sound measure of the progress in complying with national targets on the protection and enhancement of forest carbon sinks.

## Background

Forests are the largest terrestrial reservoir for atmospheric carbon. They remove CO_2 _from the atmosphere and store it in the organic matter of soil and trees. The current carbon stock in tree biomass comprises half of the atmospheric storage and is continuing to grow despite deforestation, the rate of which is decreasing but still high [1].

The amount of carbon stored in a forest stand depends on its age and productivity. The terrestrial carbon sink, inferred from changes in the concentrations of atmospheric gases and their isotopic composition, is normally attributed to the global increase in productivity [2,3]. Much less attention is paid to global changes in forest age – another important characteristic of this reservoir.

It would be too speculative to say that terrestrial carbon sink may result from the global shift in forest age, but there seems to be no escaping the conclusion that forest age is one of the points of intervention at which the future evolution of the carbon cycle might be influenced [4-7]. Most temperate and boreal forests are actively managed, and forest age depends on the length of harvest cycles there. Since biomass increases with stand age, postponing harvesting to the age of biological maturity may result in the formation of a large carbon sink [8,9].

The purpose of this article is to quantify the magnitude of the sink that may be induced by a shift in forest age on a national or regional basis.

## Results

The growth pattern of a forest stand is species and site-specific. It also depends on how the forest stand was treated at the early stage of growth. However, it is not essential to identify all of these factors in order to evaluate the effect of postponing harvesting; it is enough to know the current tree biomass (*B*_*c*_) and the age of the forest stand (*A*_*c*_).

It is shown in the Methods section that

*B*_*c *_= *P *(*A*_*c *_- *A*_1_)^4/5^   (*A*_1 _<*A*_*c *_<*A*_2_)

where *P *characterizes the productivity of a given species at a given site, *A*_1 _characterizes the response of the species to a given treatment at the early stage of stand growth, and *A*_2 _characterizes the age of biological maturity (which is also species and site-specific). Hence, postponing harvesting by Δ*A *years increases tree biomass by

ΔB=Bc[(1+ΔAAc−A1)4/5−1]≥Bc[(1+ΔAAc)4/5−1],
 MathType@MTEF@5@5@+=feaafiart1ev1aaatCvAUfKttLearuWrP9MDH5MBPbIqV92AaeXatLxBI9gBaebbnrfifHhDYfgasaacH8akY=wiFfYdH8Gipec8Eeeu0xXdbba9frFj0=OqFfea0dXdd9vqai=hGuQ8kuc9pgc9s8qqaq=dirpe0xb9q8qiLsFr0=vr0=vr0dc8meaabaqaciaacaGaaeqabaqabeGadaaakeaacqqHuoarcqWGcbGqcqGH9aqpcqWGcbGqdaWgaaWcbaGaem4yamgabeaakmaadmaabaWaaeWaaeaacqaIXaqmcqGHRaWkdaWcaaqaaiabfs5aejabdgeabbqaaiabdgeabnaaBaaaleaacqWGJbWyaeqaaOGaeyOeI0Iaemyqae0aaSbaaSqaaiabigdaXaqabaaaaaGccaGLOaGaayzkaaWaaWbaaSqabeaacqaI0aancqGGVaWlcqaI1aqnaaGccqGHsislcqaIXaqmaiaawUfacaGLDbaacqGHLjYScqWGcbGqdaWgaaWcbaGaem4yamgabeaakmaadmaabaWaaeWaaeaacqaIXaqmcqGHRaWkdaWcaaqaaiabfs5aejabdgeabbqaaiabdgeabnaaBaaaleaacqWGJbWyaeqaaaaaaOGaayjkaiaawMcaamaaCaaaleqabaGaeGinaqJaei4la8IaeGynaudaaOGaeyOeI0IaeGymaedacaGLBbGaayzxaaGaeiilaWcaaa@5999@

and the conservative estimate of the relative increase is

δB≥(1+δA)4/5−1;(δB=ΔBBc,δA=ΔAAc).
 MathType@MTEF@5@5@+=feaafiart1ev1aaatCvAUfKttLearuWrP9MDH5MBPbIqV92AaeXatLxBI9gBaebbnrfifHhDYfgasaacH8akY=wiFfYdH8Gipec8Eeeu0xXdbba9frFj0=OqFfea0dXdd9vqai=hGuQ8kuc9pgc9s8qqaq=dirpe0xb9q8qiLsFr0=vr0=vr0dc8meaabaqaciaacaGaaeqabaqabeGadaaakeaafaqabeqacaaabaacciGae8hTdqMaemOqaiKaeyyzImRaeiikaGIaeGymaeJaey4kaSIae8hTdqMaemyqaeKaeiykaKYaaWbaaSqabeaacqaI0aancqGGVaWlcqaI1aqnaaGccqGHsislcqaIXaqmcqGG7aWoaeaacqGGOaakcqWF0oazcqWGcbGqcqGH9aqpdaWcaaqaaiabfs5aejabdkeacbqaaiabdkeacnaaBaaaleaacqWGJbWyaeqaaaaakiabcYcaSiab=r7aKjabdgeabjabg2da9maalaaabaGaeuiLdqKaemyqaeeabaGaemyqae0aaSbaaSqaaiabdogaJbqabaaaaOGaeiykaKcaaiabc6caUaaa@524D@

**Figure 1:**
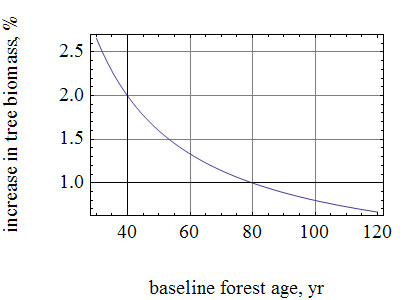
The conservative estimate of the relative increase in tree biomass (*δ B*) induced by one-year delay (Δ*A *= 1) in harvesting.

In other words, doubling the length of harvest cycle (i.e., *δ A *= 2) increases tree biomass by 40% of the baseline value. As seen in Figure 1, the annual magnitude of the sink induced by delayed harvest lies in the range of 1–2% of the baseline carbon stock.

## Discussion

Most forest stands reach their economic maturity prior to biological maturity. The size of the gap varies with tree species, site productivity, climate conditions, and the nature of the wood product. However, in general, the length of the harvest cycle could be increased at some cost [10] to create carbon sinks. The 4/5 law of forest growth gives a quick conservative estimate of the sink magnitude. For example, the tree biomass in forests of the temperate zone, most of which are managed, is estimated at 59 Gt C. Applying the 4/5 law, one may immediately conclude that postponing harvesting at 5% of the forest stands may remove 30–60 Mt C from the atmosphere per year.

From the viewpoint of forest management this application of the 4/5 law may be considered a reincarnation of Turin's principle of one point: "The normal pine stands with equal heights at a certain age had the same growth in the past and will have the same growth in the future regardless of where they grow" [11]. According to this principle, forest age is the most important indicator of the services provided by the forest ecosystems and the primary target for macro-scale management.

**Figure 2:**
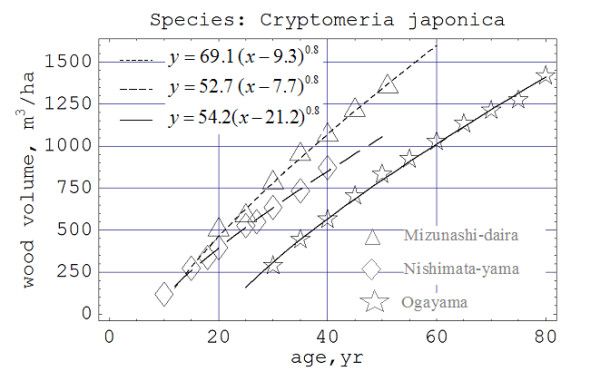
The model fits superimposed on the data from the permanent monitoring plots of the Japanese Forest Resources Monitoring System. (For restoring the normal pattern of forest growth the total amount of wood cut by a given age was added to reported wood stock at this age.)

It is well to bear in mind that Turin's principle is not always applicable on a micro scale. Tree biomass in some stands may be the same at a certain age and markedly different later (Fig 2). The effects of delayed development or intensive thinning of the forest stand at an early stage are parameterised as "rejuvenation bias" (see the Methods section), and thus micro-scale applications of the 4/5 law require two points for characterizing the growth pattern of the stand.

Thinning at an early stage of stand growth creates a variety of growth patterns that are not easy to summarize [12]. This may form the impression that the effect of postponing harvesting is difficult to assess at a national or regional scale. Indeed, one cannot assess the full extent of the effect unless the typical value of rejuvenation bias is known, but this is not required for producing a conservative estimate. Taking rejuvenation bias into account may only increase the estimates of the sink magnitude.

Moreover, it would be prudent to limit the usage of the 4/5 law with getting conservative estimates only. Forest productivity is susceptible to CO_2 _enrichment, N deposition and climate change. Recent studies show that it is increasing globally [3], and thus the 4/5 law, derived under the assumption that productivity is relatively constant, would underestimate the full extent of the sink magnitude.

Considering forest age in the context of carbon management, one should also pay attention to the fact that changes in forest age may affect carbon stocks not only in tree biomass but also in such reservoirs as forest soils and wood products. This issue is high on the agenda, but goes beyond the scope of this work.

## Conclusion

In the natural evolution of the international climate regime, the focus is switching from "what-to-do" to "how-to-do" elements of international agreements [13] and there is increased demand for monitoring the state of compliance with UN Framework Convention on Climate Change. The results of this study suggest that forest age could be one of the key measures of progress at the national or regional level. First, it is directly linked with the carbon stocks and easily understood. Second, it is measurable. Third, it raises awareness of the state of the carbon reservoir which is under direct human influence. Reporting trends in forest age would simplify quantification and communication of the progress in complying with national targets on the protection and enhancement of forest carbon sinks.

## Methods

The allometric model

*B *= *b*_1_*P A*^*c*^

linking stand age (*A*) and its biomass (*B*) at a site of known productivity (*P*) was empirically validated [14] for projecting carbon stock changes in world forests [15]. However, the theoretical validity of setting the power of age at 0.79216 is open to question. Therefore, it is worth deriving the plausible value of the power of age proceeding from some theoretical considerations, and then checking its empirical validity against available data.

Let us start from the pipe model theory of tree growth [16,17]. This theory suggests that tree biomass is proportional to the area of a stem cross section (*S*) at a height just below the joint of the lowest live branch:

*B *= *z S*

where *z *is the "Shinozaki factor".

Then, let us assume that *S *is power-law monomial of age (*A*), exergy (*E*), and the stand density (*R*) expressed in terms of biomass per volume of space occupied by the stand:

*S *= *c*_1 _*E*^*a *^*R*^*b *^*A*^*c*^

and determine the exponents, adopting the SI system of units.

In the SI units, the length (*L*) is measured in meters (m), mass (*M*) in kilograms (kg), and time (*T*) in seconds (*s*). Hence, the unit of exergy [*E*] is equal to 1 kg m^2 ^s^-1 ^– or, in other words, [*E*] = [*M*] [*L*]^2 ^[*T*]^-2^. Similarly, [*R*] = [*M*] [*L*]^-3 ^and [*A*] = [*T*].

Since [*S*] = [*E*]^*a *^[*R*]^*b *^[*A*]^*c*^,

[*L*]^2 ^= [*M*]^*a*+*b *^[L]^2*a*-3*b *^[T]^-2*a*+*c*^

so that

*a *+ *b *= 0, 2*a *- 3*b *= 2, -2*a *+ *c *= 0,

with the solution *a *= 2/5, *b *= -2/5, *c *= 4/5.

Therefore

[*S*] = [*E*]^2/5 ^[*R*]^-2/5 ^[*A*]^4/5^

and thus

*B *= *c*_1_·*z*·(*E*/*R*)^2/5 ^*A*^4/5^,

where *c*_1 _is a dimensionless coefficient.

If

*E*/*R *= const,

then biomass of a forest stand is proportional to *A*^4/5^.

The method, so-called dimensional analysis [18-20], that we used to derive the 4/5 law is a heuristic method. It is applied when finding a verifiable physical law, not for deducing it. A number of physical laws have been found in this way [21-23], however, the method itself does not guarantee success as the choice of controlling variables is of critical importance.

Our choice of controlling variables was not completely intuitive. It was based on the Jørgensen-Svirezhev theory (JST) that suggests that ecosystems are developing towards a higher level of exergy [24]. JST postulates that due to the incoming energy of solar radiation an ecosystem moves away from thermodynamic equilibrium and obtains more information and organization. This implies, among other processes, self-organization of plant canopies – that is, reducing the "randomness" of foliage distribution [25,26].

However, canopy clustering is not always a sign of increasing order. It may be a sign of decreasing order as well, as in the case of stand break-up. Therefore, we take into account the "concentration" of leaves in the space occupied by a canopy [27,28].

The ratio *E*/*R*, which reflects a relationship between the area of gaps and leaf clusters, shows whether a canopy is evolving to an ordered structure that maximizes its efficiency in terms of utilizing solar energy, or simply degrading. The biomass growth follows the 4/5 law if *E*/*R *remains constant, and that in turn suggests a certain similarity of canopy patterns in stands of different ages. Consequently, the 4/5 law can be applied only to pre-mature stands where the processes of growth and thinning are naturally balanced.

One may find a number of counterexamples to this law simply because its domain is restricted (*E*/*R *must be constant). It is not easy, however, to test whether a counterexample falls within the domain of the law or not. We may only assume that *E*/*R *is constant during a certain period of stand growth, and thus at least a part of the growth curve must fit the law. Testing the law against the data from permanent monitoring plots of the Japanese Forest Resources Monitoring System (Yield Observation Report No. 20, 1996), we also found that one cannot ignore the effect of intensive thinning. In other words, the concept of biological age [29-32] is essential for explaining variations in growth patterns induced by delayed development or intensive thinning of the forest stand at an early stage. Hence, testing the 4/5 law against observations, one should introduce a "rejuvenation bias" to take into account the difference between calendar and biological age of the stand (Fig 2).
